# An analysis of racial inequities in emergency department triage among patients with stroke-like symptoms in the United States

**DOI:** 10.1186/s12873-023-00865-z

**Published:** 2023-08-14

**Authors:** Gabriel Neves, John DeToledo, James Morris, K. Tom Xu

**Affiliations:** 1 Department of Neurology, Texas Tech University Health Sciences Center, Lubbock, TX USA; 2 Division of Emergency Medicine, Department of Surgery, Texas Tech University Health Sciences Center, Lubbock, TX USA; 3 Department of Family & Community Medicine, Texas Tech University Health Sciences Center, Lubbock, TX USA

**Keywords:** Racial inequities, Stroke, Emergency department, Physician evaluation times

## Abstract

**Background:**

Racial inequities exist in treatment and outcomes in patients with acute stroke.

**Objectives:**

Our objective was to determine if racial inequities exist in the time-lapse between patient presentation and provider assessment in patients with stroke-like symptoms in Emergency Departments (ED) across the U.S.

**Methods:**

This study is a retrospective, observational study of the National Hospital Ambulatory Medical Care Survey (NHAMCS) 2014–2018. We identified visits with stroke-like symptoms and stratified the proportion of door-to-provider (DTP) times by racial groups. We used broad and narrow definitions of stroke-like symptoms. We performed bivariate and multivariate analyses using race and clinical and demographic characteristics as covariates.

**Results:**

Between 2014–2018, there were an average of 138.58 million annual ED visits. Of the total ED visits, 0.36% to 7.39% of the ED visits presented with stroke-like symptoms, and the average DTP time ranged from 39 to 49 min. The proportion of the visits with a triage level of 1 (immediate) or 2 (emergent) ranged from 16.03% to 23.27% for stroke-like symptoms. We did not find statistically significant racial inequities in DTP or ED triage level. We found significantly longer DTP times in non-Hispanic blacks (15.88 min, 95% CI: 4.29–27.48) and Hispanics (by 14.77 min, 95% CI: 3.37–26.16) than non-Hispanic whites that presented with atypical stroke-like symptoms. We observed that non-Hispanic whites were significantly more diagnosed with a stroke/TIA than other racial minority groups (*p* = 0.045) for atypical stroke-like symptoms.

**Conclusion:**

In our population-based analysis, we did not identify systemic racial inequities in the DTP times or ED triage level at ED triage for stroke-like symptoms.

**Supplementary Information:**

The online version contains supplementary material available at 10.1186/s12873-023-00865-z.

## Article summary

### Why is this topic important?

Although stroke mortality is decreasing in the U.S., racial inequities are essential determinants of stroke disability and mortality.

### What does this study attempt to show?

Our present work assessed if racial inequities exist in the triage of patients presenting with stroke-like symptoms to ED in the U.S using a nationally representative dataset.

### What are the key findings?

Following our analysis, we did not identify systemic racial inequities in the time to initial provider assessment of these patients in the ED. We did, however, note that non-Hispanic blacks, when presenting with atypical stroke-like symptoms, had significantly greater triage times when compared to non-Hispanic whites.

### How is patient care impacted?

While our study did not reveal systemic racial inequities in the analyzed patient population, preventative strategies remain needed to increase and improve emergent healthcare access to treatment for acute stroke in the U.S.

## Introduction

For every hour of ischemia, the brain loses as many neurons as equivalent to 3.6 years of normal aging, making stroke a medical emergency [1]. Resultantly, the American Heart Association/American Stroke Association (AHA/ASA) recommends that a medical provider evaluate patients with stroke-like symptoms within 10 min of presentation to the emergency department (ED) [2]. The ED triage is a crucial component in assessing stroke patients, and their timely evaluation and treatment are key factors to maximize post-stroke recovery [3, 4].

Previous work has identified racial inequities in many domains of stroke care delivered in the United States (US) [4]. Delayed triage times and lower triage acuity levels are associated with worse stroke outcomes in racial and ethnic minorities that ultimately lead to higher stroke morbidity and mortality [5, 6]. Furthermore, compared to non-Hispanic whites, minorities less frequently receive intravenous thrombolysis and endovascular thrombectomy, the cornerstones of modern acute stroke care. When they do, it is more often delayed [7–9]. Thus, ongoing efforts to elucidate racial inequities in stroke management remain central to improving stroke outcomes for American minorities.

The ED triage is often the first point of contact for patients experiencing stroke-like symptoms. It is essential to closely examine the processes within this critical hub, where the identification of stroke-like symptoms and the initiation of prompt stroke management occur, to inform quality improvement efforts. In this study, we evaluated whether there were racial inequities in the time it took for patients to see a provider after arriving in the ED (known as door-to-provider time, or DTP) and the level of urgency assigned to them during triage among those presenting with stroke-like symptoms during ED visits in the US. We used a nationally representative dataset to conduct our analysis.

## Materials and methods

### Data

This study was a retrospective data analysis from the National Hospital Ambulatory Medical Care Survey (NHAMCS) 2014–2018 [10]. The NHAMCS is a national, multistage, sampled data set designed to characterize ED care patterns in the US. Using a three-stage probability sampling design, the NHAMCS collected information from EDs in noninstitutional general and short-stay hospitals in the 50 states and the District of Columbia. The NHAMCS randomly assigned each ED to a 4-week period during which they obtained a systematic random sample of visits. Repeat visits within 72 h, which consisted of < 5% of the visits analyzed, were not differentiated from the initial visits of the same or different chief complaints as the data set does not contain patient identifiers. Information extracted from each visit included patient characteristics, visit characteristics, diagnoses and treatments, and ED facility characteristics. The reader may find more information about the NHAMCS at the Center for Disease Control (CDC) website (http://www.cdc.gov/nchs/ahcd.htm). The NHAMCS 2014–2018 collected up to 5 chief complaints of each visit. The NHAMCS classifies each chief complaint using the Reason for Visit Classification for Ambulatory Care [11]. We combined five years of data to achieve a sufficient sample size for nationally representative estimates per the NHAMCS recommendations because of the relatively small sample size of visits associated with stroke-like symptoms; a sample < 30 or with > 30% standard error is considered unreliable [12].

### Outcome measures

We examined two widely used triage parameters as the primary outcome variables to investigate racial inequities in the ED triage process for stroke-related symptoms: the door-to-provider (DTP) time and the triage level of immediacy. We defined the DTP time as the interval between a patient's arrival time and the time of the first provider (physician or advanced practice provider) contact. The NHAMCS data categorizes the triage level of immediacy as 1 (Immediate), 2 (Emergent), 3 (Urgent), 4 (Semi-urgent), and 5 (Nonurgent), like the commonly used Emergency Severity Index (ESI) [13–15]. We dichotomized the triage level of immediacy to "Immediate or Emergent" (Levels 1 and 2) vs. non-emergent (Levels 3–5). We also examined the outcomes against a final stroke diagnosis in the ED. We identified stroke and transient ischemic attack (TIA) diagnoses based on the final diagnoses of a visit using the International Classification of Diseases (ICD 9) for 2014–2015 and ICD10 for 2016–2018: ischemic stroke (ICD9 433–434, ICD10 I630-I639), hemorrhagic stroke (ICD9 430–432, ICD10 I600-I629), and TIA (ICD9 435.9, ICD10 G45.1–45.9).

### Symptoms suggestive of potential stroke

We identified all visits between 2014–2018 in patients 18 or older with at least one chief complaint of stroke-like symptoms. We used both a narrow and a broad definition of "stroke-like symptoms" to provide reliable estimates. The narrow definition included classic stroke-like symptoms such as weakness, anesthesia/paresthesia, slurring/disorders of speech, diminished vision/blindness, half vision, and vertigo. The broad definition included all narrow definition symptoms plus non-specific or transient symptoms, such as general weakness or ill feeling, nausea, vomiting, syncope, unconsciousness, convulsions, headache, abnormal eye movement, apraxia, and dysphagia that are more likely to be misdiagnosed [16].

To further investigate whether racial inequities were present in visits with stroke-like symptoms that are less clinically obvious, we differentiated visits with chief complaints containing strictly stroke-related symptoms from visits with stroke-related symptoms intermixed with non-stroke-related symptoms, e.g., a visit with chief complaints of slurring of speech, abdominal pain, and dysuria. In total, we conducted four separate sets of analyses. The first analysis included visits if any of the five chief complaints included the symptoms under the narrow definition of symptoms suggestive of potential stroke. The second analysis included visits with all five chief complaints *recorded* only symptoms suggestive of potential stroke under the narrow definition. The third analysis included visits if any of the five chief complaints included the symptoms under the broad definition. The fourth analysis included visits with all five chief complaints *recorded* only symptoms suggestive of potential stroke under the broad definition.

### Analyses

We extracted demographic and clinical information, such as age, sex, insurance status, urban/rural area measured by Metropolitan Statistical Areas (MSAs), ambulance arrival, and triage vital signs, including heart rate, systolic blood pressure, and pulse oximeter reading. We categorized the race variable into non-Hispanic white, non-Hispanic black, Hispanic, and other races, directly corresponding to the race/ethnicity variable in the NHAMCS data collected. Notably, the estimates may not be reliable for the “other races” group in the analyses of only symptoms suggestive of potential stroke under the narrow definition, as the sample size was < 30, per NHAMCS recommendations. We excluded visits related to injury, trauma, overdose, poisoning, or adverse effect of medical/surgical treatment.

We first performed bivariate statistical analyses to examine racial inequities in the DTP time and the triage level. We examined racial inequities in the proportion of an eventual stroke diagnosis. We then performed multivariate analyses to investigate whether the racial inequities persisted after controlling for confounding sociodemographic and clinical factors, age, sex, race, insurance, urban/rural location, ambulance arrival, triage vitals and medical histories. We used logistic and linear regressions for binary and continuous dependent variables, respectively, for multivariate analyses. We used Stata (StataCorp, College Station, TX) statistical software for all analyses. We accounted for the complex sampling strategy of NHAMCS in the analysis using the "svy" commands with "subpop" in Stata. The NHAMCS used a national probability sample of visits to the ED of hospitals. It had a 4-stage probability design with samples of primary sampling units (PSU), hospitals within a PSU, EDs within a hospital, and visits within an ED. The primary sampling unit was a patient visit/encounter. The reader may find a detailed discussion of the sampling design at cdc.gov/nchs/ahcd/ahcd_scope.htm. To provide nationally representative estimates, we incorporated strata, PSUs, and sampling weights in all analyses conducted in this study. In addition, we adjusted the weights used in the analyses for five years of data to provide nationally representative annual averages.

## Results

During 2014–2018, the data set had an average of 138.58 million annual ED visits. Table 1 includes the descriptive demographic and clinical characteristics of the patient cohort of each analysis with nationally representative estimates. Females and non-Hispanic whites represented approximately 60% of the cohort. Over half of the patients within the cohort held public insurance. See Table 1 for other demographics such as race, insurance, and urban setting. The average DTP time ranged from 39 to 49 min, varying on whether the narrow or broad stroke-like symptom definition was applied. Most patients had DTP times > 10 min from the initial presentation (Table 2).

**Table 1:** Patient Characteristics (in % except for DTP time)^a^

		**Narrow Sx, Any CC**	**Narrow Sx, Only CC**	**Broad Sx, Any CC**	**Broad Sx, Only CC**
Sample size (n)		3,316	373	7,677	1,599
National estimate		4,373,693	505,694	10,242,283	2,227,674
Triage level 1 or 2		23.27	16.03	20.35	17.12
DTP time	Average minutes (S.E.)	48.60 (3.25)	39.20 (4.45)	44.03 (2.30)	39.90 (3.03)
Age	< 40	30.57	24.70	36.85	37.89
	40–64	41.25	34.69	37.06	33.43
	65 +	28.18	40.61	26.09	28.59
Sex	Male	38.44	45.99	35.99	39.53
	Female	61.56	54.01	64.01	60.47
Race	Non-Hispanic White	60.24	60.94	60.96	60.36
	Non-Hispanic black	22.01	18.55	22.50	22.74
	Hispanic	14.36	16.16	13.38	14.09
	Others	3.39	4.35	3.17	2.80
Insurance	No insurance	18.44	19.45	18.87	22.61
	Private	28.42	26.77	25.81	23.09
	Public	53.14	53.77	55.32	54.30
MSA	No	16.01	17.49	17.35	18.83
	Yes	83.99	82.51	82.65	81.17
Ambulance arrival	No	79.30	74.65	77.29	67.52
	Yes	20.70	25.35	22.71	32.48
Ambulance transfer from another facility	No	99.09	99.63	98.95	98.41
	Yes	0.91	0.37	1.05	1.59
Heart rate	60–99	75.80	75.54	72.71	72.69
	< 60	8.55	15.34	8.26	12.09
	> = 100	15.65	9.12	19.03	15.22
Systolic BP	< 140	53.29	43.11	57.33	56.45
	140–179	38.59	42.68	34.94	34.57
	> = 180	8.12	14.21	7.73	8.98
Pulse Oximeter	> = 90%	98.15	92.94	97.86	96.77
	< 90%	1.85	7.06	2.14	3.23
Prior ED visit < 72 h ago	No	96.03	95.84	95.97	96.96
	Yes	3.97	4.16	4.03	3.04
Medical Hx	Cancer	4.59	6.05	4.48	3.53
	Stroke	10.96	14.55	8.29	9.92
	Renal disease	4.85	6.08	4.65	3.19
	CHF	4.93	4.53	5.44	4.63
	CAD	13.08	14.89	10.91	10.47
	DM	18.98	21.55	17.88	17.80
	Thromboembolism	1.59	0.37	1.83	1.15
	Hyperlipidemia	18.47	16.30	15.69	15.08
	Hypertension	43.71	46.00	40.24	36.56

^a^The percentages are proportions representative of the EDs in the US, taking into account the complex sampling design

*MSA* Metropolitan Statistical Area, *Sx* Symptoms, *CC* Chief complaint, *DTP* Door-to-provider, *S.E* Standard error, *CHF* Congestive heart failure, *CAD* Coronary artery disease, *DM* Diabetes mellitus

**Table 2:** Distribution of Door-to-Provider Time by Race (%)^a^

		≤ **10 min**	**11–20 min**	**21–30 min**	**31–60 min**	**61–120 min**	** > 120 min**	***p*-value**
Narrow Sx, Any CC	NHW	29.94	18.32	9.72	14.94	10.06	17.01	0.63
	NHB	28.96	15.13	10.12	13.98	10.75	21.06	
	Hispanic	31.78	14.77	7.34	16.17	9.79	20.15	
	Others	27.71	12.60	12.09	18.98	4.99	23.62	
Narrow Sx, Only CC	NHW	31.43	9.07	8.62	15.61	8.86	26.41	0.36
	NHB	23.80	13.43	19.89	14.80	9.74	18.34	
	Hispanic	39.70	10.95	3.71	7.53	10.05	28.06	
	Others	54.12	12.42	19.71	2.65	0.00	11.10	
Broad Sx, Any CC	NHW	30.86	18.76	10.57	15.73	8.57	15.51	0.01
	NHB	28.34	17.12	9.78	13.16	10.69	20.91	
	Hispanic	30.91	14.23	6.82	17.08	11.84	19.12	
	Others	32.54	14.71	8.85	20.57	6.91	16.41	
Broad Sx, Only CC	NHW	33.85	15.76	9.22	14.74	7.54	18.89	0.03
	NHB	29.61	21.87	12.28	8.61	9.46	18.17	
	Hispanic	32.81	14.74	2.58	22.83	9.76	17.28	
	Others	39.04	11.39	11.60	14.56	1.42	21.99	

^a^ The percentages are proportions representative of the EDs in the US, taking into account the complex sampling design

*Sx* Symptoms, *CC* Chief complaint, *NHW* Non-Hispanic whites, *NHB* Non-Hispanic blacks

### Narrow definition, any of the chief complaints was stroke-related

**Fig. 1:**
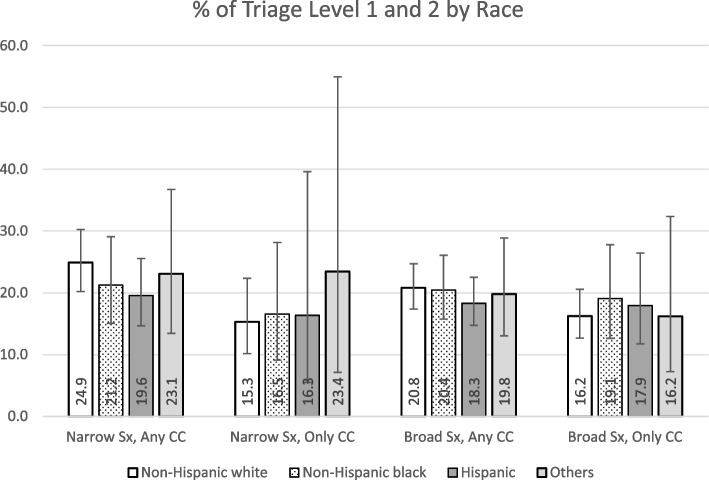
Distribution of % of visits categorized as triage level 1 and 2 by race^a^. ^a^The percentages are proportions representative of the EDs in the US, taking into account the complex sampling design, with the 95% confidence intervals presented as the whiskers

**Fig. 2:**
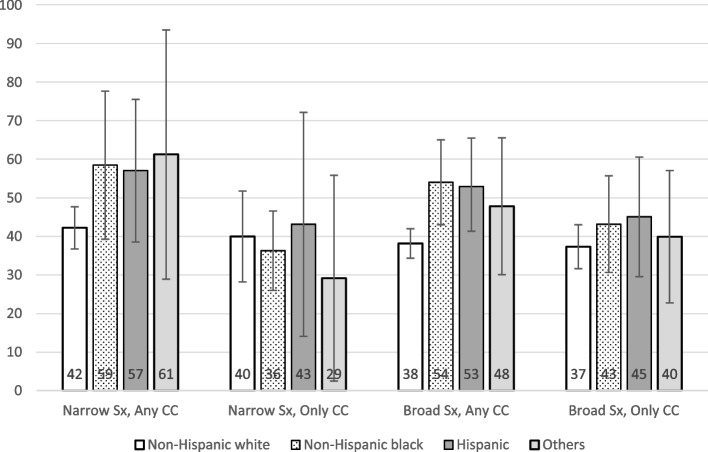
Average Door-to-Provider time (in minutes) by race^a^. ^a^ The numbers within the bars are means representative of the EDs in the US, taking into account the complex sampling design, with the 95% confidence intervals presented as the whiskers

**Fig. 3:**
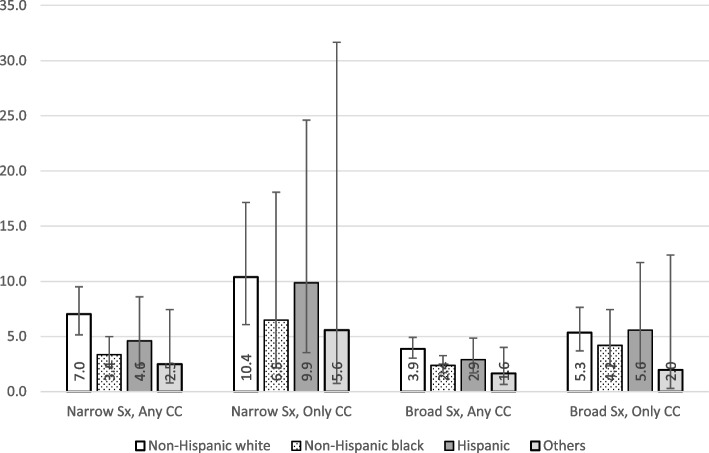
Percentage of visits with a final diagnosis of stroke or TIA by race^a^. ^a^ The percentages are proportions representative of the EDs in the US, taking into account the complex sampling design, with the 95% confidence intervals presented as the whiskers

We identified 4.37 million average annual ED visits under this category. This number of visits accounted for 3.16% of the total ED visits in the US. A triage level of 1 or 2 was present in 23.27% of the visits, with details by race/ethnicity illustrated in Fig. 1. The average DTP time was 48.60 (95% CI: 42.22–54.99) minutes, with details by race/ethnicity shown in Fig. 2. Of these visits, 29.91% had a DTP time ≤ 10 min and 19.92% had a DTP time between 11–20 min. We observed a DTP time of > 2 h in 18.58% of the visits. A diagnosis of stroke or TIA was made in 5.71% of these visits, with details by race/ethnicity shown in Fig. 3. We did not identify statistically significant racial inequities in the triage level or DTP time in the bivariate and multivariate analyses. In the multivariate analysis of triage level, age, MSA status, ambulance arrival, HR (heart rate), SBP (systolic blood pressure), prior visit, history of stroke, CHF, and hyperlipidemia were found to be statistically significant. In the multivariate analysis of DTP, HR, SBP, and history of CHF were found to be statistically significant. See Supplementary Tables 1 and 2 for details. We also found that non-Hispanic whites had significantly higher proportions of stroke diagnoses than the other groups (*p* = 0.019).

### Narrow definition, only stroke-related symptoms in chief complaints

We identified a total of 0.51 million average annual ED visits under this category, accounting for 0.36% of all ED visits in the US. A triage level 1 or 2 was present in 16.03% of the visits, as illustrated in Fig. 1. The average DTP time was 39.20 (95% CI: 30.47–47.94) minutes, as shown in Fig. 2. Of these visits, 32.34% had DTP time ≤ 10 min and 10.33% had DTP time between 11–20 min. We observed a DTP time of > 2 h in 24.51% of the visits. A diagnosis of stroke or TIA in the ED was associated with 9.36% of these visits, as shown in Fig. 3. Bivariate and multivariate analyses did not demonstrate statistically significant racial inequities in the triage level, DTP time, or diagnosis of stroke/TIA. In the multivariate analysis of triage level, age, HR, oxygenation, history of stroke were statistically significant. In the multivariate analysis of DTP, history of cancer was found to be statistically significant. See Supplementary Tables 3 and 4 for details.

### Broad definition, any of the chief complaints was stroke-related

We identified a total of 10.24 million average annual ED visits under this category, which accounted for 7.39% of the total ED visits in the US. A triage level 1 or 2 was present in 20.35% of the visits, as illustrated in Fig. 1. The average DTP time was 44.03 (95% CI: 39.51–48.56) minutes, as shown in Fig. 2. Of these visits, 30.35% had DTP time ≤ 10 min and 17.66% had DTP time between 11–20 min. We observed a DTP time of > 2 h in 17.24% of the visits. A diagnosis of stroke or TIA was present in 3.32% of these visits, as shown in Fig. 3. We did not identify statistically significant racial inequities at the triage level in both bivariate and multivariate analyses. In the multivariate analysis of triage level, age, sex, MSA status, ambulance arrival, HR, SBP, prior visit, history of stroke and history of hyperlipidemia were statistically significant. See Supplementary Table 5 for details. However, non-Hispanic blacks had a statistically significantly longer DTP time with a difference of 15.88 min (54.07 vs. 38.19 min; *p* = 0.007, 95% CI: 4.29–27.48) when compared to non-Hispanic whites. Hispanics also had a significantly longer DTP time than non-Hispanic whites by 14.77 min (*p* = 0.011, 95% CI: 3.37–26.16). In the multivariate analyses, controlling for other sociodemographic characteristics and clinical factors, non-Hispanic blacks had an average of 11.97 min longer DTP time than non-Hispanic whites (*p* = 0.030, 95% CI: 1.17–22.77), and Hispanics had an average of 11.84 min longer DTP time than non-Hispanic whites (*p* = 0.042, 95% CI: 0.43–23.24). Other statistically significant variables in the multivariate analysis of DTP included MSA status, ambulance arrival, HR, SBP, oxygenation, and history of renal disease. See Supplementary Table 6 for details. Lastly, non-Hispanic whites were significantly more frequently diagnosed with a stroke/TIA than other racial and ethnic minority groups (*p* = 0.045) in the bivariate analysis. The stroke/TIA diagnosis proportions for non-Hispanic white, non-Hispanic black, Hispanic, and others were 3.86%, 2.37%, 2.88%, and 1.64%, respectively.

### Broad definition, only stroke-related symptoms in chief complaints

We identified a total of 2.23 million average annual ED visits under this category, which accounted for 1.61% of the total ED visits in the US. A triage level of 1 or 2 was present in 17.12% of the visits, as illustrated in Fig. 1. The average DTP time was 39.90 (95% CI: 33.95–45.85) minutes, as shown in Fig. 2. Of these visits, 32.89% had DTP time ≤ 10 min and 16.88% had DTP time between 11–20 min. We observed a DTP time of > 2 h in 18.59% of the visits. A diagnosis of stroke or TIA was present in 5.01% of these visits, as shown in Fig. 3. We did not identify statistically significant racial inequities in the triage level or DTP time in the bivariate or multivariate analyses. In the multivariate analysis of triage level, ambulance arrival, HR, oxygenation, and history of stroke were found to be statistically significant. In the multivariate analysis of DTP, MSA status, ambulance arrival, SBP, oxygenation, and history of renal disease and thromboembolism were statistically significant. See Supplementary Tables 7 and 8 for details. There were no racial/ethnic differences in the mean DTP. The differences in the distribution, however, were statistically significant (*p* = 0.03), as shown in Table 2.

## Discussion

This observational study did not find significant systemic racial inequities in ED triage of patients presenting with stroke-like symptoms. However, we identified differences between visits with atypical stroke-like symptoms and non-stroke-related symptoms. Previous work using the NHAMCS database found racial inequities in ischemic stroke patients' ED waiting times [6]. This study analyzed a small number of visits only with a confirmed stroke diagnosis and not stroke-like symptoms. The present study aimed to represent real-life scenarios as patients often present with stroke-like symptoms, as only a fraction of patients with stroke-like symptoms has a final stroke diagnosis. Previous literature demonstrated that non-Hispanic blacks are more likely to receive delayed intravenous thrombolysis and endovascular thrombectomy, which are associated with worse overall stroke outcomes [5–8]. Prior findings suggest non-Hispanic blacks receive delayed attention for symptoms suggestive of potential stroke because they often present to higher volume centers that experience more ED crowding or have a delayed presentation [17–19]. However, we did not test this hypothesis directly in our study as the measurement of ED crowding was not available in the secondary data set.

A noteworthy finding in our study was that a healthcare provider failed to readily assess approximately 2/3 of patients presenting with stroke-like symptoms to US EDs within the recommended 10 min from presentation, per guidelines from the AHA/ASA [2]. This finding persisted independent of the stroke symptom category (e.g., narrow or broad). Surprisingly, approximately 20% of patients presenting with stroke-like symptoms wait > 120 min to be assessed by an ED provider in some centers. In a prospective single-center analysis, an ED provider's median delay to assessment was 0.42 h, including patients arriving by emergency medical services (EMS) [15]. Our observation agrees with prior studies and indicates that quality improvement efforts are still needed to accelerate the triage of patients with symptoms suggestive of potential stroke [8, 20].

Previous studies showed that in the general population, non-Hispanic blacks, independent of gender, have a three-fold more significant risk of stroke when compared with non-Hispanic whites [21, 22]. However, among patients presenting to ED with symptoms suggestive of stroke, which is more pertinent to triage and ED physicians than patients in the community, our results suggest that racial and ethnic minorities may be less frequently diagnosed with stroke than non-Hispanic whites in the ED. Our finding is consistent with a previous large study of a national cohort that demonstrated that a higher proportion of non-Hispanic blacks had stroke-like symptoms without a stroke diagnosis when compared to non-Hispanic whites [23]. In addition, previous work has demonstrated that stroke in non-Hispanic whites is more often misdiagnosed [24, 25]. In a multi-regional study, non-Hispanic blacks were 18% more likely to be misdiagnosed with stroke than non-Hispanic whites [24]. However, we could not exclude that stroke was underdiagnosed in non-Hispanic blacks as this group received less thorough diagnostic evaluation or presented more frequently with atypical symptoms suggestive of a potential stroke, such as headache or dizziness [24, 26].

Like stroke, longer door-to-intervention times result in worse outcomes for ST-elevation myocardial infarction [27]. Although racial inequities in the overall incidence of acute coronary syndrome (ACS) have narrowed, some racial inequities persist in ACS; further, women presenting to the ED with ST-segment elevation myocardial infection (STEMI) are significantly less likely to receive door-to-activation time within the AHA guidelines then are men [28, 29]. Analogous to our findings, patients with atypical chest pain presentations appear more likely to suffer delays in their care [30]. Overall, these observed delays noted in the acute care of patients presenting to the ED with ACS may be mitigated by protocols that recognize chief complaints associated with atypical chest pain, such as shortness of breath, or by the placement of a provider in triage, reducing DTP time [31, 32]. Similar strategies could improve the triage of patients presenting with stroke-like symptoms.

### Limitations

This study has several limitations due to secondary data and cross-sectional study design. It is retrospective; thus, the observed associations do not imply causation. Coding accuracy may also impact the validity of the data we analyzed, as the NHAMCS is a sizeable secondary dataset. In addition, our dataset lacks follow-up and outcome data, which precludes analysis of the clinical outcomes of the identified associations. Furthermore, the data needed information regarding whether a nurse, physician or advanced practitioner triaged a patient. Consequently, we could not explore differences by provider type at triage. Lastly, the NHAMCS database does not contain clinical information, such as symptom onset, symptom severity, and comprehensive stroke center designation. Therefore, the generalizability of our work is limited.

## Conclusion

We did not identify systemic racial inequities in ED triage level and DTP times in patients with stroke-like symptoms in US EDs. However, non-Hispanic blacks and Hispanics, when presenting with atypical stroke-like symptoms mixed with non-stroke-related symptoms, experienced longer DTP times when compared to non-Hispanic whites. Significantly, EDs across the US must improve triage processes to expedite the triage process for patients presenting with stroke-like symptoms, as ED providers delayed beyond the recommended 10 min the initial evaluation on more than 2/3 of patients in our cohort, per the AHA/ASA guidelines.

### Supplementary Information

**Additional file 1: Supplementary Table 1.** Multivariate analysis results of high triage levels (1 or 2) for narrow definition, stroke-related symptoms in any of the chief complaints. **Supplementary Table 2.** Multivariate analysis results of door-to-provider time for narrow definition, stroke-related symptoms in any of the chief complaints. **Supplementary Table 3.** Multivariate analysis results of high triage levels (1 or 2) for narrow definition, only stroke-related symptoms in the chief complaints. **Supplementary Table 4.** Multivariate analysis results of door-to-provider time for narrow definition, only stroke-related symptoms in the chief complaints. **Supplementary Table 5.** Multivariate analysis results of high triage levels (1 or 2) for broad definition, stroke-related symptoms in any of the chief complaints. **Supplementary Table 6.** Multivariate analysis results of door-to-provider time for broad definition, stroke-related symptoms in any of the chief complaints. **Supplementary Table 7.** Multivariate analysis results of high triage levels (1 or 2) for broad definition, only stroke-related symptoms in the chief complaints.

## Data Availability

The data used for this study was obtained from a public domain, CDC’s website, http:// https://www.cdc.gov/nchs/ahcd/datasets_documentation_related.htm.
