# Supplementary material for: An analysis of racial inequities in emergency department triage among patients with stroke-like symptoms in the United States
Source: BMC Emerg Med. 2023 Aug 14;23:90. doi: 10.1186/s12873-023-00865-z (PMC10426180; doi:10.1186/s12873-023-00865-z)
Supplement: Supplementary file 1 — Additional file 1: Supplementary Table 1. Multivariate analysis results of high triage levels (1 or 2) for narrow definition, stroke-related symptoms in any of the chief complaints. Supplementary Table 2. Multivariate analysis results of door-to-provider time for narrow definition, stroke-related symptoms in any of the chief complaints. Supplementary Table 3. Multivariate analysis results of high triage levels (1 or 2) for narrow definition, only stroke-related symptoms in the chief complaints. Supplementary Table 4. Multivariate analysis results of door-to-provider time for narrow definition, only stroke-related symptoms in the chief complaints. Supplementary Table 5. Multivariate analysis results of high triage levels (1 or 2) for broad definition, stroke-related symptoms in any of the chief complaints. Supplementary Table 6. Multivariate analysis results of door-to-provider time for broad definition, stroke-related symptoms in any of the chief complaints. Supplementary Table 7. Multivariate analysis results of high triage levels (1 or 2) for broad definition, only stroke-related symptoms in the chief complaints. [file 12873_2023_865_MOESM1_ESM.docx]

Supplementary Table 1 Multivariate analysis results of high triage levels (1 or 2) for narrow definition, stroke-related symptoms in any of the chief complaints

|  |  | OR | p | 95% CI |
| --- | --- | --- | --- | --- |
| Age | <40 |  |  |  |
|  | 40-64 | 1.754 | 0.000 | (1.295, 2.377) |
|  | 65+ | 1.971 | 0.016 | (1.133, 3.427) |
| Sex | Male |  |  |  |
|  | Female | 0.872 | 0.256 | (0.687, 1.105) |
| Race | Non-Hispanic White | |  |  |
|  | Non-Hispanic black | 0.906 | 0.665 | (0.577, 1.421) |
|  | Hispanic | 0.799 | 0.325 | (0.511, 1.250) |
|  | Others | 0.817 | 0.602 | (0.382, 1.748) |
| Insurance | No insurance |  |  |  |
|  | Private | 1.006 | 0.979 | (0.658, 1.537) |
|  | Public | 0.903 | 0.602 | (0.615, 1.326) |
| MSA | No |  |  |  |
|  | Yes | 1.831 | 0.034 | (1.047, 3.203) |
| Ambulance arrival | No |  |  |  |
|  | Yes | 1.370 | 0.019 | (1.053, 1.782) |
| Ambulance transfer from another facility | No |  |  |  |
|  | Yes | 5.813 | 0.001 | (1.968, 17.173) |
| Heart rate | 60-99 |  |  |  |
|  | <60 | 1.430 | 0.179 | (0.848, 2.411) |
|  | >=100 | 1.458 | 0.025 | (1.049, 2.027) |
| Systolic BP | <140 |  |  |  |
|  | 140-179 | 1.090 | 0.449 | (0.872, 1.362) |
|  | >=180 | 1.970 | 0.001 | (1.339, 2.899) |
| Pulse Oximeter | >=90% |  |  |  |
|  | <90% | 0.888 | 0.841 | (0.277, 2.848) |
| Prior ED visit <72 hrs ago | No |  |  |  |
|  | Yes | 0.303 | 0.006 | (0.129, 0.712) |
| Medical Hx | Cancer | 1.179 | 0.525 | (0.709, 1.961) |
|  | Stroke | 2.706 | 0.000 | (1.955, 3.746) |
|  | Renal disease | 0.819 | 0.457 | (0.483, 1.388) |
|  | CHF | 2.262 | 0.013 | (1.187, 4.311) |
|  | CAD | 1.155 | 0.478 | (0.775, 1.723) |
|  | DM | 1.040 | 0.820 | (0.743, 1.455) |
|  | Thromboembolism | 0.447 | 0.086 | (0.178, 1.123) |
|  | Hyperlipidemia | 1.632 | 0.002 | (1.194, 2.231) |
|  | Hypertension | 0.945 | 0.724 | (0.688, 1.296) |

Supplementary Table 2 Multivariate analysis results of door-to-provider time for narrow definition, stroke-related symptoms in any of the chief complaints

|  |  | beta | p | 95% CI |
| --- | --- | --- | --- | --- |
| Age | <40 |  |  |  |
|  | 40-64 | -4.562 | 0.662 | (-25.047, 15.923) |
|  | 65+ | -8.400 | 0.539 | (-35.262, 18.463) |
| Sex | Male |  |  |  |
|  | Female | 6.348 | 0.150 | (-2.309, 15.006) |
| Race | Non-Hispanic White | |  |  |
|  | Non-Hispanic black | 10.479 | 0.206 | (-5.767, 26.725) |
|  | Hispanic | 11.939 | 0.243 | (-8.107, 31.985) |
|  | Others | 18.100 | 0.277 | (-14.557, 50.757) |
| Insurance | No insurance |  |  |  |
|  | Private | 6.438 | 0.367 | (-7.559, 20.436) |
|  | Public | 7.214 | 0.283 | (-5.973, 20.402) |
| MSA | No |  |  |  |
|  | Yes | 7.560 | 0.438 | (-11.574, 26.695) |
| Ambulance arrival | No |  |  |  |
|  | Yes | -4.544 | 0.538 | (-19.024, 9.935) |
| Ambulance transfer from another facility | No |  |  |  |
|  | Yes | -9.380 | 0.575 | (-42.187, 23.427) |
| Heart rate | 60-99 |  |  |  |
|  | <60 | -9.147 | 0.169 | (-22.207, 3.913) |
|  | >=100 | -14.777 | 0.001 | (-23.152, 6.402) |
| Systolic BP | <140 |  |  |  |
|  | 140-179 | -0.074 | 0.992 | (-15.104, 14.957) |
|  | >=180 | -16.206 | 0.032 | (-31.034, 1.379) |
| Pulse Oximeter | >=90% |  |  |  |
|  | <90% | -7.821 | 0.577 | (-35.364, 19.722) |
| Prior ED visit <72 hrs ago | No |  |  |  |
|  | Yes | 28.870 | 0.452 | (-46.486, 104.226) |
| Medical Hx | Cancer | -14.158 | 0.062 | (-29.017, 0.701) |
|  | Stroke | -13.629 | 0.090 | (-29.389, 2.131) |
|  | Renal disease | -12.234 | 0.144 | (-28.649, 4.181) |
|  | CHF | -21.069 | 0.012 | (-37.444, 4.695) |
|  | CAD | 8.012 | 0.546 | (-18.049, 34.073) |
|  | DM | 10.313 | 0.270 | (-8.025, 28.651) |
|  | Thromboembolism | 7.390 | 0.618 | (-21.693, 36.474) |
|  | Hyperlipidemia | 16.243 | 0.135 | (-5.086, 37.572) |
|  | Hypertension | 5.880 | 0.186 | (-2.842, 14.602) |

Supplementary Table 3 Multivariate analysis results of high triage levels (1 or 2) for narrow definition, only stroke-related symptoms in the chief complaints

|  |  | OR | p | 95% CI |
| --- | --- | --- | --- | --- |
| Age | <40 |  |  |  |
|  | 40-64 | 6.003 | 0.019 | (1.341, 26.873) |
|  | 65+ | 3.714 | 0.104 | (0.765, 18.036) |
| Sex | Male |  |  |  |
|  | Female | 1.319 | 0.585 | (0.488, 3.571) |
| Race | Non-Hispanic White | |  |  |
|  | Non-Hispanic black | 1.032 | 0.959 | (0.315, 3.377) |
|  | Hispanic | 0.869 | 0.855 | (0.192, 3.934) |
|  | Others | 2.315 | 0.291 | (0.487, 11.002) |
| Insurance | No insurance |  |  |  |
|  | Private | 0.440 | 0.371 | (0.073, 2.665) |
|  | Public | 0.560 | 0.344 | (0.168, 1.865) |
| MSA | No |  |  |  |
|  | Yes | 0.751 | 0.678 | (0.194, 2.905) |
| Ambulance arrival | No |  |  |  |
|  | Yes | 1.140 | 0.802 | (0.407, 3.197) |
| Ambulance transfer from another facility | No |  |  |  |
|  | Yes* |  |  |  |
| Heart rate | 60-99 |  |  |  |
|  | <60 | 4.040 | 0.018 | (1.273, 12.823) |
|  | >=100 | 0.906 | 0.897 | (0.202, 4.059) |
| Systolic BP | <140 |  |  |  |
|  | 140-179 | 0.385 | 0.089 | (0.128, 1.159) |
|  | >=180 | 0.547 | 0.339 | (0.159, 1.887) |
| Pulse Oximeter | >=90% |  |  |  |
|  | <90% | 0.012 | 0.009 | (0.000, 0.329) |
| Prior ED visit <72 hrs ago | No |  |  |  |
|  | Yes | 0.647 | 0.627 | (0.111, 3.765) |
| Medical Hx | Cancer | 1.065 | 0.943 | (0.187, 6.068) |
|  | Stroke | 6.728 | 0.000 | (2.444, 18.517) |
|  | Renal disease | 0.543 | 0.386 | (0.136, 2.165) |
|  | CHF | 1.637 | 0.548 | (0.327, 8.180) |
|  | CAD | 1.946 | 0.210 | (0.686, 5.526) |
|  | DM | 1.126 | 0.823 | (0.395, 3.208) |
|  | Thromboembolism* |  |  |  |
|  | Hyperlipidemia | 1.352 | 0.493 | (0.570, 3.211) |
|  | Hypertension | 0.908 | 0.844 | (0.347, 2.378) |

*: perfect prediction without any variation of the outcome.

Supplementary Table 4 Multivariate analysis results of door-to-provider time for narrow definition, only stroke-related symptoms in the chief complaints

|  |  | beta | p | 95% CI |
| --- | --- | --- | --- | --- |
| Age | <40 |  |  |  |
|  | 40-64 | 15.252 | 0.372 | (-18.303, 48.806) |
|  | 65+ | -14.164 | 0.174 | (-34.607, 6.279) |
| Sex | Male |  |  |  |
|  | Female | -10.161 | 0.358 | (-31.873, 11.551) |
| Race | Non-Hispanic White | |  |  |
|  | Non-Hispanic black | -5.840 | 0.587 | (-26.949, 15.268) |
|  | Hispanic | -4.153 | 0.815 | (-38.998, 30.693) |
|  | Others | -1.832 | 0.925 | (-40.222, 36.558) |
| Insurance | No insurance |  |  |  |
|  | Private | 2.312 | 0.834 | (-19.369, 23.992) |
|  | Public | 20.557 | 0.278 | (-16.618, 57.733) |
| MSA | No |  |  |  |
|  | Yes | 12.662 | 0.244 | (-8.648, 33.971) |
| Ambulance arrival | No |  |  |  |
|  | Yes | -18.103 | 0.064 | (-37.248, 1.043) |
| Ambulance transfer from another facility | No |  |  |  |
|  | Yes | 0.460 | 0.977 | (-31.062, 31.982) |
| Heart rate | 60-99 |  |  |  |
|  | <60 | -5.322 | 0.729 | (-35.497, 24.852) |
|  | >=100 | 7.601 | 0.512 | (-15.141, 30.343) |
| Systolic BP | <140 |  |  |  |
|  | 140-179 | -10.470 | 0.355 | (-32.667, 11.728) |
|  | >=180 | -10.216 | 0.363 | (-32.237, 11.806) |
| Pulse Oximeter | >=90% |  |  |  |
|  | <90% | -23.944 | 0.155 | (-56.969, 9.081) |
| Prior ED visit <72 hrs ago | No |  |  |  |
|  | Yes | -15.070 | 0.342 | (-46.187, 16.047) |
| Medical Hx | Cancer | -17.473 | 0.032 | (-33.457, -1.488) |
|  | Stroke | -13.339 | 0.147 | (-31.362, 4.684) |
|  | Renal disease | -15.163 | 0.135 | (-35.075, 4.749) |
|  | CHF | 30.281 | 0.119 | (-7.765, 68.327) |
|  | CAD | 9.900 | 0.281 | (-8.119, 27.919) |
|  | DM | 1.376 | 0.854 | (-13.269, 16.021) |
|  | Thromboembolism | 7.713 | 0.883 | (-95.042, 110.469) |
|  | Hyperlipidemia | -5.620 | 0.560 | (-24.544, 13.305) |
|  | Hypertension | -13.069 | 0.186 | (-32.438, 6.301) |

*: perfect prediction without any variation of the outcome.

Supplementary Table 5 Multivariate analysis results of high triage levels (1 or 2) for broad definition, stroke-related symptoms in any of the chief complaints

|  |  | OR | p | 95% CI |
| --- | --- | --- | --- | --- |
| Age | <40 |  |  |  |
|  | 40-64 | 1.663 | 0.000 | (1.293, 2.138) |
|  | 65+ | 1.911 | 0.004 | (1.238, 2.949) |
| Sex | Male |  |  |  |
|  | Female | 0.808 | 0.030 | (0.666, 0.979) |
| Race | Non-Hispanic White | |  |  |
|  | Non-Hispanic black | 1.085 | 0.667 | (0.748, 1.574) |
|  | Hispanic | 1.020 | 0.906 | (0.727, 1.432) |
|  | Others | 0.892 | 0.702 | (0.497, 1.602) |
| Insurance | No insurance |  |  |  |
|  | Private | 1.066 | 0.668 | (0.795, 1.429) |
|  | Public | 0.945 | 0.658 | (0.736, 1.213) |
| MSA | No |  |  |  |
|  | Yes | 2.122 | 0.005 | (1.256, 3.585) |
| Ambulance arrival | No |  |  |  |
|  | Yes | 1.557 | 0.000 | (1.259, 1.927) |
| Ambulance transfer from another facility | No |  |  |  |
|  | Yes | 2.604 | 0.008 | (1.291, 5.251) |
| Heart rate | 60-99 |  |  |  |
|  | <60 | 1.284 | 0.182 | (0.889, 1.855) |
|  | >=100 | 1.381 | 0.002 | (1.131, 1.687) |
| Systolic BP | <140 |  |  |  |
|  | 140-179 | 1.037 | 0.676 | (0.874, 1.230) |
|  | >=180 | 1.714 | 0.002 | (1.213, 2.421) |
| Pulse Oximeter | >=90% |  |  |  |
|  | <90% | 1.142 | 0.706 | (0.572, 2.282) |
| Prior ED visit <72 hrs ago | No |  |  |  |
|  | Yes | 0.544 | 0.020 | (0.326, 0.907) |
| Medical Hx | Cancer | 1.090 | 0.623 | (0.772, 1.539) |
|  | Stroke | 2.308 | 0.000 | (1.819, 2.928) |
|  | Renal disease | 1.208 | 0.219 | (0.893, 1.634) |
|  | CHF | 1.442 | 0.058 | (0.988, 2.105) |
|  | CAD | 1.338 | 0.061 | (0.987, 1.814) |
|  | DM | 1.207 | 0.110 | (0.958, 1.521) |
|  | Thromboembolism | 1.134 | 0.613 | (0.696, 1.848) |
|  | Hyperlipidemia | 1.737 | 0.000 | (1.330, 2.269) |
|  | Hypertension | 1.033 | 0.754 | (0.841, 1.271) |

Supplementary Table 6 Multivariate analysis results of door-to-provider time for broad definition, stroke-related symptoms in any of the chief complaints

|  |  | beta | p | 95% CI |
| --- | --- | --- | --- | --- |
| Age | <40 |  |  |  |
|  | 40-64 | -1.029 | 0.848 | (-11.545, 9.488) |
|  | 65+ | -6.376 | 0.344 | (-19.596, 6.844) |
| Sex | Male |  |  |  |
|  | Female | 2.871 | 0.288 | (-2.434, 8.177) |
| Race | Non-Hispanic White | |  |  |
|  | Non-Hispanic black | 11.970 | 0.030 | (1.169, 22.771) |
|  | Hispanic | 11.837 | 0.042 | (0.428, 23.247) |
|  | Others | 6.955 | 0.436 | (-10.556, 24.466) |
| Insurance | No insurance |  |  |  |
|  | Private | 0.985 | 0.830 | (-8.027, 9.996) |
|  | Public | 5.412 | 0.247 | (-3.754, 14.578) |
| MSA | No |  |  |  |
|  | Yes | 10.872 | 0.040 | (0.494, 21.251) |
| Ambulance arrival | No |  |  |  |
|  | Yes | -12.035 | 0.000 | (-18.188, -5.883) |
| Ambulance transfer from another facility | No |  |  |  |
|  | Yes | -3.844 | 0.574 | (-17.269, 9.582) |
| Heart rate | 60-99 |  |  |  |
|  | <60 | -4.732 | 0.239 | (-12.611, 3.147) |
|  | >=100 | -7.642 | 0.003 | (-12.708, -2.576) |
| Systolic BP | <140 |  |  |  |
|  | 140-179 | -0.448 | 0.895 | (-7.138, 6.242) |
|  | >=180 | -8.958 | 0.023 | (-16.657, -1.258) |
| Pulse Oximeter | >=90% |  |  |  |
|  | <90% | -12.301 | 0.038 | (-23.893, -0.710) |
| Prior ED visit <72 hrs ago | No |  |  |  |
|  | Yes | 15.602 | 0.395 | (-20.378, 51.582) |
| Medical Hx | Cancer | -5.804 | 0.221 | (-15.107, 3.498) |
|  | Stroke | -3.350 | 0.567 | (-14.839, 8.138) |
|  | Renal disease | -10.590 | 0.037 | (-20.555, -0.625) |
|  | CHF | -1.389 | 0.786 | (-11.448, 8.670) |
|  | CAD | 1.375 | 0.874 | (-15.631, 18.381) |
|  | DM | 4.182 | 0.358 | (-4.751, 13.116) |
|  | Thromboembolism | 1.439 | 0.869 | (-15.643, 18.520) |
|  | Hyperlipidemia | 7.352 | 0.246 | (-5.077, 19.781) |
|  | Hypertension | 4.487 | 0.169 | (-1.914, 10.888) |

Supplementary Table 7 Multivariate analysis results of high triage levels (1 or 2) for broad definition, only stroke-related symptoms in the chief complaints

|  |  | OR | p | 95% CI |
| --- | --- | --- | --- | --- |
| Age | <40 |  |  |  |
|  | 40-64 | 1.540 | 0.174 | (0.826, 2.870) |
|  | 65+ | 1.584 | 0.239 | (0.736, 3.408) |
| Sex | Male |  |  |  |
|  | Female | 0.788 | 0.202 | (0.546, 1.137) |
| Race | Non-Hispanic White | |  |  |
|  | Non-Hispanic black | 1.099 | 0.723 | (0.651, 1.856) |
|  | Hispanic | 1.108 | 0.763 | (0.568, 2.160) |
|  | Others | 0.963 | 0.950 | (0.302, 3.072) |
| Insurance | No insurance |  |  |  |
|  | Private | 0.623 | 0.204 | (0.301, 1.293) |
|  | Public | 0.821 | 0.506 | (0.459, 1.470) |
| MSA | No |  |  |  |
|  | Yes | 1.921 | 0.099 | (0.884, 4.174) |
| Ambulance arrival | No |  |  |  |
|  | Yes | 1.803 | 0.012 | (1.142, 2.847) |
| Ambulance transfer from another facility | No |  |  |  |
|  | Yes | 2.189 | 0.200 | (0.660, 7.257) |
| Heart rate | 60-99 |  |  |  |
|  | <60 | 2.132 | 0.040 | (1.036, 4.389) |
|  | >=100 | 1.128 | 0.648 | (0.672, 1.894) |
| Systolic BP | <140 |  |  |  |
|  | 140-179 | 0.776 | 0.256 | (0.500, 1.203) |
|  | >=180 | 0.977 | 0.946 | (0.504, 1.893) |
| Pulse Oximeter | >=90% |  |  |  |
|  | <90% | 0.170 | 0.009 | (0.045, 0.645) |
| Prior ED visit <72 hrs ago | No |  |  |  |
|  | Yes | 0.264 | 0.057 | (0.067, 1.043) |
| Medical Hx | Cancer | 0.979 | 0.959 | (0.427, 2.243) |
|  | Stroke | 3.597 | 0.000 | (2.151, 6.016) |
|  | Renal disease | 1.533 | 0.303 | (0.679, 3.461) |
|  | CHF | 0.947 | 0.875 | (0.478, 1.875) |
|  | CAD | 0.931 | 0.823 | (0.496, 1.746) |
|  | DM | 1.403 | 0.167 | (0.867, 2.270) |
|  | Thromboembolism | 1.191 | 0.823 | (0.257, 5.533) |
|  | Hyperlipidemia | 1.264 | 0.337 | (0.784, 2.038) |
|  | Hypertension | 1.326 | 0.233 | (0.834, 2.107) |

Supplementary Table 8 Multivariate analysis results of door-to-provider time for broad definition, only stroke-related symptoms in the chief complaints

|  |  | beta | p | 95% CI |
| --- | --- | --- | --- | --- |
| Age | <40 |  |  |  |
|  | 40-64 | 8.065 | 0.225 | (-4.987, 21.117) |
|  | 65+ | 2.833 | 0.730 | (-13.255, 18.920) |
| Sex | Male |  |  |  |
|  | Female | -4.349 | 0.406 | (-14.619, 5.921) |
| Race | Non-Hispanic White | |  |  |
|  | Non-Hispanic black | 7.224 | 0.288 | (-6.117, 20.565) |
|  | Hispanic | 5.025 | 0.527 | (-10.565, 20.615) |
|  | Others | 3.214 | 0.739 | (-15.743, 22.171) |
| Insurance | No insurance |  |  |  |
|  | Private | -0.386 | 0.959 | (-15.018, 14.247) |
|  | Public | -0.637 | 0.912 | (-11.913, 10.639) |
| MSA | No |  |  |  |
|  | Yes | 16.624 | 0.002 | (5.933, 27.316) |
| Ambulance arrival | No |  |  |  |
|  | Yes | -16.838 | 0.000 | (-25.001, -8.676) |
| Ambulance transfer from another facility | No |  |  |  |
|  | Yes | 11.596 | 0.432 | (-17.402, 40.593) |
| Heart rate | 60-99 |  |  |  |
|  | <60 | 3.755 | 0.639 | (-11.964, 19.474) |
|  | >=100 | 7.539 | 0.248 | (-5.254, 20.332) |
| Systolic BP | <140 |  |  |  |
|  | 140-179 | -10.075 | 0.070 | (-20.958, 0.808) |
|  | >=180 | -10.727 | 0.141 | (-25.032, 3.578) |
| Pulse Oximeter | >=90% |  |  |  |
|  | <90% | -26.010 | 0.002 | (-42.454, 9.566) |
| Prior ED visit <72 hrs ago | No |  |  |  |
|  | Yes | 11.648 | 0.625 | (-35.179, 58.475) |
| Medical Hx | Cancer | -3.252 | 0.674 | (-18.449, 11.945) |
|  | Stroke | -0.688 | 0.953 | (-23.686, 22.310) |
|  | Renal disease | -21.535 | 0.011 | (-38.087, 4.982) |
|  | CHF | 12.075 | 0.196 | (-6.261, 30.411) |
|  | CAD | 0.390 | 0.957 | (-13.892, 14.672) |
|  | DM | 2.019 | 0.730 | (-9.467, 13.504) |
|  | Thromboembolism | -22.797 | 0.028 | (-43.168, 2.425) |
|  | Hyperlipidemia | -8.306 | 0.289 | (-23.681, 7.069) |
|  | Hypertension | 6.958 | 0.216 | (-4.066, 17.982) |
